# Effects of Wolves on Elk and Cattle Behaviors: Implications for Livestock Production and Wolf Conservation

**DOI:** 10.1371/journal.pone.0011954

**Published:** 2010-08-04

**Authors:** Isabelle Laporte, Tyler B. Muhly, Justin A. Pitt, Mike Alexander, Marco Musiani

**Affiliations:** 1 Faculty of Environmental Design, University of Calgary, Calgary, Alberta, Canada; 2 Department of Biological Sciences, University of Alberta, Alberta, Canada; 3 Lands Division, Alberta Sustainable Resource Development, Pincher Creek, Alberta, Canada; University of Pretoria, South Africa

## Abstract

**Background:**

In many areas, livestock are grazed within wolf (*Canis lupus*) range. Predation and harassment of livestock by wolves creates conflict and is a significant challenge for wolf conservation. Wild prey, such as elk (*Cervus elaphus*), perform anti-predator behaviors. Artificial selection of cattle (*Bos taurus*) might have resulted in attenuation or absence of anti-predator responses, or in erratic and inconsistent responses. Regardless, such responses might have implications on stress and fitness.

**Methodology/Principal Findings:**

We compared elk and cattle anti-predator responses to wolves in southwest Alberta, Canada within home ranges and livestock pastures, respectively. We deployed satellite- and GPS-telemetry collars on wolves, elk, and cattle (n = 16, 10 and 78, respectively) and measured seven prey response variables during periods of wolf presence and absence (speed, path sinuosity, time spent head-up, distance to neighboring animals, terrain ruggedness, slope and distance to forest). During independent periods of wolf presence (n = 72), individual elk increased path sinuosity (Z = −2.720, *P* = 0.007) and used more rugged terrain (Z = −2.856, *P* = 0.004) and steeper slopes (Z = −3.065, *P* = 0.002). For cattle, individual as well as group behavioral analyses were feasible and these indicated increased path sinuosity (Z = −2.720, *P* = 0.007) and decreased distance to neighbors (Z = −2.551, *P* = 0.011). In addition, cattle groups showed a number of behavioral changes concomitant to wolf visits, with variable direction in changes.

**Conclusions/Significance:**

Our results suggest both elk and cattle modify their behavior in relation to wolf presence, with potential energetic costs. Our study does not allow evaluating the efficacy of anti-predator behaviors, but indicates that artificial selection did not result in their absence in cattle. The costs of wolf predation on livestock are often compensated considering just the market value of the animal killed. However, society might consider refunding some additional costs (e.g., weight loss and reduced reproduction) that might be associated with the changes in cattle behaviors that we documented.

## Introduction

In many areas of the world, livestock are grazed within wolf (*Canis lupus*) range. Predation and harassment of domestic animals by wolves creates conflict with humans and is a significant challenge for wolf conservation and management in those regions [1]. Beyond the direct effect of predation (i.e., death), the presence of wolves in close proximity to livestock may cause prey to change their behavior to avoid predation, called a risk [2], non-consumptive [3] or trait-mediated [4] effect of predators. While anti-predator behaviors in response to wolves are documented in wild prey such as elk (*Cervus elaphus*) (e.g., [5]), these are relatively unknown in free-ranging large ungulate livestock [6], [7]. Both domestic and wild animals may respond to predators with adaptive behaviors such as increased vigilance [8]–[10], grouping and changes in group sizes [11]–[13], changes in habitat selection [5], [7], and various changes in movement patterns [14]–[17]. However, domestic prey often show weaker responses than wild animals [18], [19] because of unfamiliarity with predators, artificial selection by humans, and they are typically kept in enclosures further limiting the options available to perform anti-predator responses compared to free-ranging wild prey [20]–[22]. Anti-predator behaviors, if present, might result in increased stress [23], which might make cattle more vulnerable to infections and diseases [24], abortion and early birth [25], and weight loss of adults [26]. The risk effects of wolves on livestock might therefore ultimately influence human tolerance for wolves in livestock production areas.

We tested for anti-predator responses for a typical wild prey and a typical domestic prey species of wolves that are sympatric in southwest Alberta, Canada (elk and cattle, respectively) [7], [27], [28]. The aim of our study was to improve our knowledge of wild and domestic prey anti-predator responses to wolves and ascertain the potential for presence of non-consumptive effects of predators on cattle that may be costly for livestock producers.

We used satellite- and GPS-telemetry technology to identify wolf presence in elk home ranges and cattle pastures. We analyzed the following behaviors in elk and cattle comparing periods of wolf presence to periods before and after such visits: movements (speed and sinuosity), vigilance (using time spent head-up as index), tendency to group, and habitat use patterns. Overall, we assessed whether elk and cattle responded to wolf presence. We also documented with what type of behavior and when each ungulate species responded. We predicted a response by cattle to wolf presence because we hypothesized that their anti-predator responses were present, although perhaps attenuated due to domestication and artificial selection by humans. We predicted elk would perform anti-predator responses to wolves, because wild prey necessitates such responses for reasons of fitness. We found that both elk and cattle modify their behavior in relation to wolf presence, with unexpected variable direction in changes for cattle and with the potential for energetic costs in both species.

## Materials and Methods

### Ethics Statement

The wolf, elk and cattle capturing, handling and monitoring protocols for this research were reviewed and approved by the Universities of Alberta and Calgary and by all jurisdictions of the Alberta Government (Permit Numbers: BI-2008-19, RC-06SW-001 and 23181CN). All animal use followed the guidelines established by the Canadian Council on Animal Care.

### Study area

**Figure 1:**
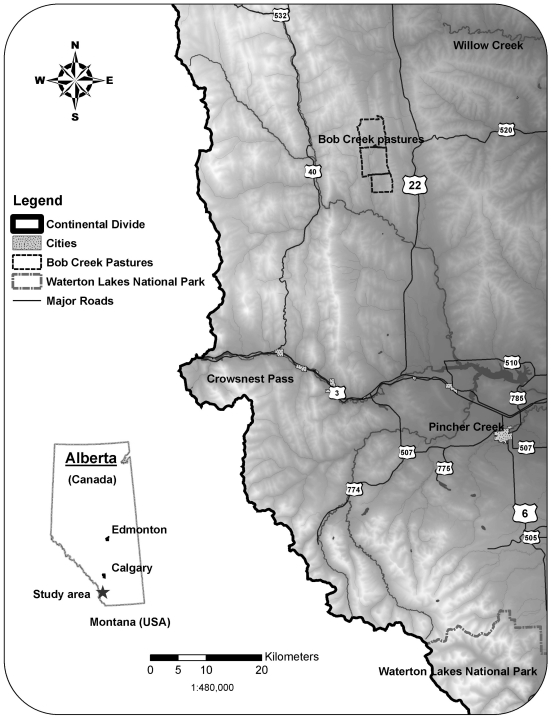
Map of the study area in southwest Alberta, Canada. The Bob Creek Pastures where cattle were GPS-collared, as well as major towns and highways are indicated.

Our study area was located in southwestern Alberta, Canada along the eastern slopes of the Rocky Mountains (Fig. 1). It is delineated by Waterton Lakes National Park at the southern edge and by Willow Creek to the north. The western boundary is the Alberta/British-Columbia border and the eastern boundary is Highway 22 and Highway 6. Wild prey for wolves in the area include elk, white-tailed deer (*Odocoileus virginianus*), mule deer (*O. hemionus*), and moose (*Alces alces*). Cattle are the predominant domestic herbivore, but domestic sheep (*Ovis aries*) also occur in a few areas. The livestock industry is an important economic activity for residents. The economy of the area is composed of agriculture, forestry and oil and gas development. The dominating land use is domestic livestock grazing, mostly cattle, which takes place both on public and private lands [29].

### Cattle, elk, and wolf locations

Cattle, elk, and wolf locations were collected using satellite and GPS-telemetry technologies. Cattle location data came from GPS-collared (Lotek 3300L) heifers and steers kept in a forest reserve grazing allotment located in the Bob Creek Wildland (Fig. 1) composed of three pastures, named Beaverdam (12 km^2^), Buck (23 km^2^), and Bailey (23 km^2^). This grazing allotment was managed by Alberta Public Lands and Forests, Alberta Sustainable Resource Development (ASRD) division [30] and was representative of other grazing dispositions in southwest Alberta. Public land grazing allotments are typically high-wolf-predation risk areas because of their forested and remote locations [29]. Location data were collected over three successive years from 2004 to 2006 in the three different pastures. In 2004, from 1 July to 14 September, nine GPS-telemetry collars were deployed with a 20 minute fix interval. In 2005, from 1 April to 1 May and from 1 July to 10 September, eleven collars were deployed with a 10 minute fix interval, except between midnight to 5 am when fix the interval was 30 minutes. The same sampling design was employed in 2006; however, no data were collected from 1 April to 1 May. A total of 249,115 locations were collected using GPS-telemetry collars, with an average location error of 37 m. Of those, 3,078 locations occurred within the pre-phase, treatment phase and post-phase and were thus used in analysis.

Elk location data came from 22 GPS-telemetry collared (Lotek 4400L and 2200L) cows during the period of 12 January to 15 October, 2007. The fix frequency was set at two hour intervals. We collected a total of 56,362 locations from the GPS-telemetry collars, with an average location error of 39 m. Of those, 1,377 locations occurred within the pre-phase, treatment phase and post-phase and were used in analysis.

From 2003 to 2007, thirty-six wolves from four different packs were captured and fitted with ARGOS satellite-telemetry collars, and at least one wolf per pack was collared each year. Locations of wolf pack members are generally spatially cohesive [31] therefore a sample of one wolf from each pack each year yielded the minimum needs of our study. We obtained 8,172 wolf locations with a minimum location accuracy of 1,000 m according to the Location Quality Index of the ARGOS collars. The average fix interval was 9 hours.

### Determining wolf presence and absence periods

**Figure 2:**
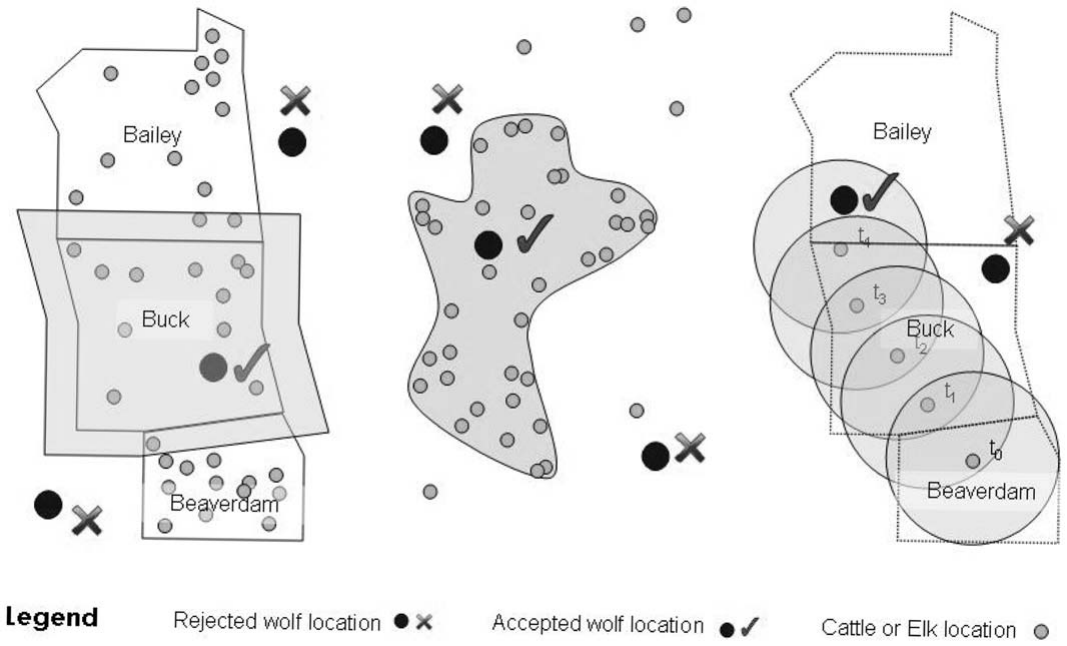
Illustration of the methodology used to define periods of known wolf presence (i.e., the treatment phase) in proximity to GPS-collared prey (locations indicated by small gray circles). Treatment phases were the periods when wolf satellite-telemetry locations (large black circles) occurred in cattle pastures (left), elk home ranges (middle) or within a 1.5 km buffer of a cattle location (right).

Our approach required a clear definition of wolf presence, that is, when and where wolves were likely detectable by elk or cattle. Because cattle were confined to pastures, we identified wolf presence periods when a wolf was located within the cattle pasture and a surrounding buffer of 1.5 km (Fig. 2). We included this buffer as a conservative means to account for detection distance of predators by prey. A detection distance of one to two kilometers has been assumed to identify short-term predation risk response by prey in other studies of large mammal predator-prey interactions [13], [32]. According to Creel et al. [13], prey can detect predators within a watershed at a scale of 32 km^2^.

We also developed a parallel method to determine wolf presence periods in very close proximity to cattle. We identified wolf locations within a 1.5 km radius of cattle locations, as opposed to the pasture (Fig. 2). If a wolf occurred within 1.5 km of a cattle location, then the wolf was considered detectable. The 1.5 km-buffer method offers a less conservative alternative for identifying wolf-cattle interactions compared to the cattle pasture method outlined above.

Elk are free-ranging animals therefore we defined wolf presence periods when a wolf occurred within an elk home range. For each individual elk, we estimated a winter and summer home range, as elk on the eastern slopes of the Rocky Mountains use different ranges during winter and summer [33]. We defined the winter season as 12 January to 31 May and the summer season as 1 June to 13 October, comparable to previous studies on elk [13], [33]. We estimated elk home ranges using a 95% kernel density estimator [34] with a smoothing parameter (*h* = 3 km) determined based on our knowledge of elk distribution in the study area. We did not buffer the home ranges because visual inspection of the kernels indicated that the 95% contour extended one to two kilometers outside the actual telemetry locations, a distance comparable to the buffer we used around cattle pastures. To identify all wolf presence and absence periods we automated queries in Geographic Information Systems (GIS) and in Microsoft Access software.

The temporal precision of our wolf location data (one location every nine hours) made it difficult to determine exactly when a wolf entered and departed elk home ranges and cattle pastures. As a conservative means to account for this uncertainty we added to the wolf presence period 4.5 hours (i.e. half of the average duration between locations) before and 4.5 hours after the time wolves occurred in the elk home range or cattle pasture. Thus the minimum treatment phase was nine hours, and longer if >1 consecutive wolf location occurred in the home range or pasture.

Behavioral studies have shown that there may be a lag in prey responses to predators resulting in a post-treatment phase different from the pre-treatment phase [35] therefore we included a post-treatment phase. The pre-phase and post-phase were defined as 18-hour periods before and after the treatment phase, because 18 hours was the average length of all treatment phases. Uniform pre-phase and post-phase durations were used to ensure experimental phases were consistent and thus comparable to each other. Furthermore, an 18-hour period was appropriate to minimize the effects of daily patterns in animal behavior on the results. Patterns in animal behavior may occur over a 24-hour cycle [36]. These daily patterns occur in wolves [37] and elk [38] and may occur in cattle too. Thus, tests comparing elk and cattle behaviors occurring in shorter time-frames may show significant differences that are simply due to changes in daily patterns in activity - i.e. not due to a wolf visit.

### Measuring prey response to wolves

We compared prey behaviors performed by individual elk or cattle between phases using a matched-pair design [39]. Each animal was considered independently, i.e., the individual was the sampling unit. This is a pragmatic approach to account for the potential influence of autocorrelation and unbalanced sample sizes of GPS location data collected from different animals on the outcome of the analysis [40], [41]. From a statistical perspective, as long as the interval between locations is constant in the three phases of our analysis, biases resulting from autocorrelation issues should be taken care of, or accounted for equally. In our study, we used GPS locations collected with the same sampling interval and technology. Further, we found our data to be consistent among the three phases.

To detect significant changes in prey behavior we used non-parametric tests because these tests are well established in animal behavioral studies as being robust in reaching various assumptions and are suitable for small sample sizes [39]. We used a Friedman test to test the null hypothesis that the three phases were drawn from the same population (i.e., behaviors during the three phases are similar). If the null hypothesis was rejected (i.e., phases were different), then we used a two-tailed Wilcoxon test to assess which phase was different from the others. If a significant difference was detected between the pre-treatment and the treatment phase, we considered that the prey had reacted to wolf presence. We also compared the behavior of groups of cattle between each phase with the same matched-pair design using the cattle pasture and 1.5 km buffer method for defining wolf presence.

### Prey response variables: movement and environmental metrics

Because anti-predator behaviors are a composite of many responses which an animal can adjust to accomplish its end, we calculated seven prey response variables to characterize cattle and elk responses to wolf presence (Table S1). These variables were movement and environmental metrics calculated directly from GPS-telemetry and habitat data that are typically considered in studies of animal anti-predator responses. We calculated the speed, sinuosity, time spent head-up, distance to neighbors, terrain ruggedness, slope and distance to forest cover for each cattle and elk observation associated with each pre-treatment, treatment, and post-treatment phase of wolf events. We did not calculate time spent head-up and distance to neighbors for elk because the elk GPS-telemetry collars did not have head-up/head-down sensors and we did not have >1 elk with a GPS -telemetry collar in the same herd during wolf presence, respectively.

When prey detect predators they may run away (the “flight” response) to avoid a direct encounter with a predator and thus speed of prey may increase during periods of wolf presence. Speed is defined as the distance traveled by the animal per unit time [42]. For our study, we defined the unit of time as the phase duration (Table S1; Eq. 1). Sinuosity, also called path tortuousity, is a simple and sensitive way to characterize the straightness of an animal's path [43]. It is the ratio between the net displacement and the path length (Table S1; Eq. 2), where the net displacement refers to the straight line between the starting and ending locations of a path [42]. Values can range from 0 to 1, where 0 indicates a path close to a straight line. A straight path may indicate a flight response, as the animal leaves an area to avoid a predator. Conversely, a high sinuosity pathway may indicate foraging or grouping behavior. For example, an animal foraging intensively on a productive patch of food may move slowly but with much turning.

We calculated the distance to neighbors to test if individual cattle were scattered or grouped during each phase. Many behavioral studies have indicated that herding animals, such as elk and cattle, will group together for the benefits of collective vigilance and defense, and dilution of risk [12], [44]. We computed this metric using equation 3 (Table S1), for cattle only.

We calculated the time with head up for each cattle using the head activity data from the cattle GPS collars (Table S1, Eq.4). Each GPS collar was equipped with head activity sensors that provide information on the animal's head movement along the X, Y, and Z axes. A value is derived from the true tilt-switch sensor and estimates the percentage of time the animal's head was up during a predefined sampling period [45]. In this case, the head activity sampling period was fixed at 5 minute intervals. Information gained from head activity sensors cannot be directly correlated with animal behaviors, especially without field validations [45]. Thus, our ability to make behavioral inferences from this variable was limited. However, we may infer general non-grazing behaviors which may include scanning, travelling, grooming, and conspecific interactions [44]–[47].

Terrain affects the grazing and travelling behavior of prey species such as cattle and elk [48]–[50]. Rugged terrain might also provide security cover [16]. We calculated terrain ruggedness [51] (Table S1, Eq. 5) in GIS from a digital elevation model (DEM) with a 30-m^2^ spatial resolution. Because slope also affects the distribution of cattle and elk on the landscape [52], [53], we included slope in our environmental metrics. We derived the percent slope (Table S1, Eq. 6) from the same DEM using Spatial Analyst in ArcGIS 9.2. We calculated slope and terrain ruggedness values for each cattle and elk location using the Intersect Point tool in Hawth's Tools v.3.26 in ArcGIS 9.2.

Some studies have reported that in the presence of a predator, elk or cattle may move into the protective cover of forested areas [5], [7]. To test for this anti-predator response, we calculated the distance to forest cover of each elk and cattle location. We defined forest security cover for ungulates as forested areas with a canopy closure above 75% [54] and using a GIS model of canopy closure [55] we calculated the distance of each animal location to forest cover with 75% canopy closure. We extracted the distance to forest cover values for each cattle and elk location using the Intersect Point tool in Hawth's Tools v.3.26.

Analyses feasible for cattle and elk datasets were different, limiting the ability to compare results. Results obtained for cattle in pastures could be compared with those obtained for elk within their home ranges. However, we could not analyze changes in groups of elk behavior nor could we acquire data on elk distance to neighbors because we only had one elk collared in a home range per event, unlike cattle enclosed as groups in a pasture. Furthermore, we could not calculate time with head up for elk due to difference in collar design.

## Results

### Cattle Responded To Wolf Presence with Variable Behavioral Changes

We identified 19 independent wolf visits to cattle pastures and 8 independent events in which wolves occurred within a 1.5 km buffer of an individual cattle location. One confirmed wolf depredation on cattle occurred within the study pasture during the study period, suggesting cattle were responding to wolves as predators, not just as novel stimuli. No wolf visits to cattle pastures were measured in 2006. Results obtained using the cattle pasture vs. the 1.5 km-buffer methodological approaches were analyzed separately in this paper.

Across all individual cattle monitored in cattle pastures, we found differences in cattle path sinuosity and distance to neighbor between wolf pre-treatment, treatment (i.e. wolf visit proper) and post-treatment phases (χ^2^ = 7.103, *P* = 0.029; χ^2^ = 6.727, *P* = 0.035; Table 1). Specifically, sinuosity increased from the pre-treatment to the treatment phase as cattle zigzagged more (Z = −2.720, *P* = 0.007), and decreased from the treatment to post-treatment phase (Z = −3.220, *P* = 0.001). Distance to neighbor decreased from the pre-treatment to the treatment phase as cattle grouped (Z = −2.551, *P* = 0.011), and also from the treatment to the post-treatment phase (Z = −2.112, *P* = 0.035). Thus, distance to neighbor obviously decreased from pre-treatment to post-treatment, indicating a lasting effect (Z  = −3.054, *P* = 0.002).

**Table 1:** Across all individuals, elk and cattle consistently changed some of their behavior in response to wolf presence (treatment phase) compared to wolf absence (pre- treatment and post- treatment phases) within elk home ranges and cattle pastures in southwest Alberta, Canada during 2004–2007.

Metric^1^ Species	Movement rate	Head up	Path sinuosity	Distance to neighbors	Terrain ruggedness index	Slope	Distance to forest cover	Number of wolf visits
Wolf within cattle pasture	NC^2^	NC	↑^3^	↓	NC	NC	NC	78
Wolf within elk home range	NC	N/A	↑	N/A	↑	↑	NC	72

1 See Table S1 for details on how each metric of prey behavior was calculated.

2 Insignificant Wilcoxon test's (No Change).

3 Arrow indicates a significant difference between the paired pre-phase and treatment phase (assessed using a Wilcoxon test) and the direction of the change.

With cattle enclosed in pastures, we could also analyze consistency of behavior between groups of cattle across wolf visit events (Table 2). Groups in cattle pastures also changed their behaviors; however, the direction of these changes was variable. Of the 19 wolf visits to cattle pastures, seven prompted at least one change in a prey response variable (6.000<χ^2^<18.200, 0.001<*P*<0.05). However, the type and direction of the response behavior, and the phase during which the observed behavior changed, both varied among events. For instance, focusing only on behaviors that are comparable to elk (see below), the sinuosity of cattle movements increased for event two and decreased for event seven between pre-phase and treatment-phase. On the other hand, in event fourteen, sinuosity decreased, but only in the post-phase, indicating a lagged response. Terrain ruggedness of cattle habitat increased for event sixteen and decreased for event four between pre- and treatment- phases. As a final example, slope of cattle habitat increased for event sixteen between pre- and treatment- phases. However, in event four, slope decreased only in the post-phase, also indicating a lagged response.

**Table 2:** Groups of cattle within pastures erratically changed their behavior in response to wolf presence (treatment phase) compared to wolf absence (pre- treatment and post- treatment phases) in cattle pastures in southwest Alberta, Canada during 2004–2005.

Event	Wolf visit (date)	Movement rate	Head up	Path sinuosity	Distance to neighbors	Terrain ruggedness index	Slope	Distance to forest cover	Cattle monitored (n)
1	06 Jul 2004	NC^1^	↑^2^	NC	NC	NC	NC	NC	5
2	26 Jul 2004	NC	↑	↑	↑	NC	NC	↓	5
3	18 Apr 2005	↓	↑↑^3^	NC	↓↓	NC	NC	NC	10
4	06 Apr 2005	↑↑	NC	NC	NC	↓	↓↓	NC	11
5	02 Jul 2005	NC	NC	NC	NC	NC	NC	NC	4
6	19 Jul 2005	NC	NC	NC	NC	NC	NC	NC	4
7	19 Jul 2005	↓	NC	↓	NC	NC	NC	NC	7
8	30 Jul 2005	NC	NC	NC	NC	NC	NC	NC	3
9	31 Jul 2005	NC	NC	NC	NC	NC	NC	NC	3
10	02 Aug 2005	NC	NC	NC	NC	NC	NC	NC	3
11	03 Aug 2005	NC	NC	NC	NC	NC	NC	NC	3
12	24 Aug 2005	NC	NC	NC	NC	NC	NC	NC	3
13	26 Aug 2005	NC	NC	NC	NC	NC	NC	NC	3
14	26 Aug 2005	↑	NC	↓↓	↓	NC	NC	NC	6
15	01 Sep 2005	NC	NC	NC	NC	NC	NC	NC	3
16	01 Sep 2005	↑	NC	NC	↓	↑	↑	NC	6
17	02 Sep 2005	NC	NC	NC	NC	NC	NC	NC	3
18	03 Sep 2005	NC	NC	NC	NC	NC	NC	NC	3
19	09 Sep 2005	NC	NC	NC	NC	NC	NC	NC	3

1 Insignificant across individuals Wilcoxon test's (No Change).

2 Single arrows indicate a significant difference between the paired pre- and treatment phases (assessed using a Wilcoxon test) and the direction of the change.

3 Double arrows indicate a significant difference between the paired treatment and post-phases (assessed using a Wilcoxon test) and the direction of the change.

For cattle, we could also analyze consistency of behavior across wolf visit events by groups in the same pasture using a 1.5 km-buffer methodological approach (Fig. 1). When wolves approached within 1.5 km of an individual cattle, there was a behavioral change in the herd in four out of eight events (6.000<χ^2^<10.000,0.007<P<0.050; Table 3). However, similar to the cattle pasture analysis (above), the direction of these changes was highly variable, with no clear pattern in response across events.

**Table 3:** Groups of cattle within pastures erratically changed their behavior in response to wolf presence (treatment phase) compared to wolf absence (pre- treatment and post- treatment phases) within a 1.5 km buffer of individual cattle locations in southwest Alberta, Canada during 2004–2005.

Wolf visit (date)	Movement rate	Head up	Path sinuosity	Distance to neighbors	Terrain ruggedness index	Slope	Distance to forest cover	Cattle monitored (n)
06 Jul 2004	NC^1^	NC	NC	NC	NC	NC	NC	4
26 Jul 2004	NC	↑^2^	↑	↑	NC	NC	↓	5
06 Apr 2005	↑↑	NC	NC	NC	↓↓^3^	NC	NC	10
18 Apr 2005	↓	↑	NC	↓↓	NC	NC	↓	5
03 Jul 2005	NC	NC	NC	NC	NC	NC	NC	4
19 Jul 2005	NC	NC	NC	NC	NC	NC	NC	4
26 Aug 2005	NC	NC	NC	NC	NC	NC	NC	4
1^st^ Sep 2005	NC	NC	NC	NC	↑↑	↑↑	NC	4

1 Insignificant across individuals Wilcoxon test's (No Change).

2 Single arrows indicate a significant difference between the paired pre- and treatment phases (assessed using a Wilcoxon test) and the direction of the change.

3 Double arrows indicate a significant difference between the paired treatment and post-phases (assessed using a Wilcoxon test) and the direction of the change.

### Elk Used Steeper Slope, Rugged Terrain and Increased Path Sinuosity in Response to Wolf Presence

Monitored wolves frequented individual elk home ranges on 72 occasions. We could document differences between path sinuosity, terrain ruggedness, and slope during pre-treatment, treatment, and post-treatment phases (6.333<χ^2^<6.861, 0.032<*P*<0.042; Table 1). Slope (Z = −3.065, *P* = 0.002) and terrain ruggedness (Z = −2.856, *P* = 0.004) of habitat were higher during the treatment phase compared to both the pre-treatment phase and the post-treatment phase (Z = −2.329, P = 0.020; Z = −2.351, *P* = 0.019), as is expected in wild prey species. Consistent with the latter changes, path sinuosity increased between the pre-treatment and the treatment phases (Z = −2.664, *P* = 0.008).

## Discussion

### Cattle Responded Variably To Wolf Visits

Overall, in this study cattle behaviorally responded to wolf visits consistently and coincidentally with patterns described by Muhly et al. [7] for habitat selection analyses conducted on the same dataset. Concordance of findings of these two studies supports the notion that cattle perform anti-predator behaviors. However, despite the prediction that cattle would respond to wolves (see above), in both studies such responses were variable.

In particular, cattle responded to wolf presence in the pasture or in a 1.5 km buffer around individual cattle locations (Tables 1, 2 and 3). However, such responses were variable in type and direction (Tables 2 and 3), and as a result anti-predator behaviors of cattle seemed erratic. This study as well as the analysis by Muhly et al. [7] both suggests that cattle may lack consistent, predictable or prompt anti-predator behaviors. In fact, we would expect cattle to establish a consistent and immediate response if they were capable of effective anti-predator behaviors. It should be mentioned here that “good” anti-predator behaviors are not needed for the majority of cattle, which are kept in farms where efficient predators are not present. As a result, anti-predator behaviors may not be under selection in cattle, and may actually be selected against (see below). Finally, we detected changes despite that certain environmental characteristics (e.g., presence of fences) might have limited the range of behavioral changes in cattle by restricting movements or available habitat types.

Our findings that cattle moved closer to other cattle and increased path sinuosity (Table 1) may suggest that cattle form groups in the presence of wolves. Grouping is used by prey to dilute predation risk among individuals in the group and increase group vigilance (the many-eyes hypothesis [56]) so prey have more time to spend grazing [11]–[13], [57], [58]. Grouping is also a common strategy in some large wild bovids [59] and cattle may have maintained a similar instinctual, anti-predator behavior despite domestication. The instinctual grouping behavior of cattle in relation to disturbance may be advantageous for livestock producers as they often herd cattle into groups to move them around the landscape. Many livestock producers also use domestic dogs to herd cattle, which cattle might relate to as to wolves [60]. Our inference on presence and importance of anti-predator behaviors in cattle has to be limited. In our study, responses of groups of cattle to wolf visits were highly variable (Tables 2 and 3). Changes in prey response variables were detected, but inconsistent across events.

Variability in anti-predator responses is found also in wild prey. A study conducted on captive wild elk in Alberta concluded that elk strongly react to predator odours, but observed significant variability in the reactions [61]. Lind and Cresswell [62] have argued that prey adopt a range of behaviors at different stages of the predation event to effectively balance cost and benefits of each type of defense strategy, depending on the predation scenario. Anti-predator strategies also depend on landscape and predator hunting mode circumstances [63], for example if prey is chased or stalked. It is still possible that the high degree of variability in anti-predator behavior that we found in cattle indicates adoption of specific anti-predator strategies, depending on the situation. However, other non-adaptive explanations of such variability seem more likely.

Other studies of livestock responses to disturbances, such as fear [64], flight speed [65], restrain vs. open [66], and novelty tests [67] have found a high degree of variability in cattle responses. These studies suggested that such variability might be due to the loss of anti-predator traits in cattle as a result of the domestication and artificial selection processes. Cattle were likely domesticated by humans 10,000 years ago [68]. Since then, humans have been selectively breeding animals that were tamer and less aggressive [20]. Tameness has likely been selected for to facilitate animal handing and to maximize weight gain, as tamer animals might lose less energy in movements [69]. As a result, humans may have ultimately selected for such traits at the expense of anti-predator behaviors.

Finally, cattle might show variability in responses because of lack of experience with predators when compared to wild prey species that live with predators year-round. In addition, in our study monitored cattle were yearling animals that had been separated from their mothers (females are called ‘heifers’ and males are called ‘steers’ by ranchers). Young cattle might have even less experience with predator visits, nor can they follow older individuals with more experience and perhaps a clearer action plan. Thus, naivety of young livestock in our study may have further contributed to variability in responses.

### Elk respond to wolf visits by moving to steeper areas

Our results show that, similar to cattle, elk also reacted to wolf presence; however, elk increased their use of steep slope and rugged terrain as well as their pathway sinuosity. The use of rugged terrain and steep slope by elk as refuge from predation is concordant with what found for elk in other studies [13], but not in domestic cattle monitored by us. Such response is typical in wild prey and is considered an efficient anti-predator response [70], [71]. Contrary to cattle, our methodological approach did not allow us to infer on elk grouping behavior and its relationship to increased sinuosity (see above). However, sinuosity is expected to increase if elk move to steeper, more rugged terrain as in this study, and this too might be a common pattern of prey reactions.

Overall, we observed that cattle, like elk, increased their pathway sinuosity; whereas elk, unlike cattle, also significantly increased their use of rugged terrain and steeper slopes. Thus, anti-predator strategies of elk appear to be more habitat-based than those of cattle. This difference may be also due to elk having more options available from which to select their habitat relative to cattle. The range of habitats available to cattle to select from (i.e., forests, slopes and rugged terrain) and the ability to flee from predators (i.e., move in straight line) might have been limited by fencing. However, the range of habitats in pastures was typical of the study area, and cattle pastures were of comparable size to elk home ranges and encompassed a similar array of habitats [7]. Regardless, all behavioral changes detected for elk and cattle in the presence of wolves imply increased energetic costs.

### Implications for cattle production and wolf conservation

The variable behavioral responses to predators that we found in cattle might have fitness costs. For example, predation can increase the stress levels of animals and result in reduced reproduction [72]. In elk, reduced reproduction due to predation from wolves appears to be mediated by elk changing their foraging pattern in response to wolves (which has nutritional costs) rather than by increasing stress levels [73]. Lind and Cresswell [62] suggested that where the cost of anti-predation behavior is higher, as in naïve prey for example, then we may reasonably conclude that anti-predation behavior affects health and fitness of prey. A similar logic might be applied to our cattle, for which we could reject the null hypothesis of no behavioral responses, and thus no risk effects of wolves on livestock.

Reduced weight gain and reproduction as well as injury caused by predator harassment of livestock are concerns of many ranchers [22] and thus may reduce their tolerance for large carnivores, including wolves. Our results suggest wolves influence cattle behaviour but we did not measure subsequent fitness costs (whether mediated by stress, changes in forage intake, or something else). Therefore indentifying the presence of a risk effect mechanism and whether it has consequences to animal fitness (e.g., reduced weight gain and reproduction) is an important area of future research, as it might help quantify economic consequences for livestock producers.

The frequency of wolf presence events might also influence the potential consequences of predation risk effects on cattle. During some periods of our study visits by wolves to pastures were infrequent (e.g., we measured no visits in 2006) therefore fitness consequences for cattle were unlikely. However, frequent visits during some periods of this study (e.g., approximately every week in July and August 2005, see Table 2) are more likely to have fitness consequences to cattle that are economically costly for livestock producers. The fitness consequences as a function of the frequency of interaction between wolves and cattle also warrants further consideration in future research, as livestock producers whose herds experience more frequent visits from wolves might suffer greater economic consequences.

Financial compensation for livestock killed by wolves is a tool used to promote tolerance for wolves in many areas where wolves kill livestock [1]. However, many current compensation programs for livestock depredation by wolves only compensate for the costs of direct predator effects (i.e., killed livestock) [74], [75]. In most jurisdictions behavioral effects of wolf presence on livestock are not officially acknowledged, so no compensation is provided for these. The effectiveness of compensation programs is debated [76], however, they could potentially be improved by compensating for the non-consumptive effects of wolves on prey, such as those that we documented and could not quantify. Farmers and ranchers have always been convinced that such additional costs (e.g., lower value of cattle due to reduced weight gain and reproduction) exist and should be considered by compensation programs. While it is very difficult to measure and thus compensate the actual financial costs of wolf presence [75], compensation payments could be based on predation risk in addition to actual kills to at least acknowledge the potential non-consumptive effects of wolves on prey fitness. In some areas of the world, this approach is being employed [77], [78]. In addition, as predation risk can be estimated using a probability model for livestock kills [29], we also suggest that livestock producers could receive a higher compensation amount in higher risk areas. Ultimately, if both predator conservation and livestock production are objectives in the same area, compensation for non-consumptive effects is worthy of consideration.

## Supporting Information

Table S1 Equations and illustrations of the habitat and movement metrics calculated from elk and cattle GPS-telemetry data during periods of wolf presence (treatment phase) and absence (pre- and post-phases) in home ranges and pastures, respectively, in southwest Alberta Canada.
(0.16 MB PDF)
