# Supplementary material for: Effects of Wolves on Elk and Cattle Behaviors: Implications for Livestock Production and Wolf Conservation
Source: PLoS One. 2010 Aug 4;5(8):e11954. doi: 10.1371/journal.pone.0011954 (PMC2915913; doi:10.1371/journal.pone.0011954)
Supplement: Table S1 — Equations and illustrations of the habitat and movement metrics calculated from elk and cattle GPS-telemetry data during periods of wolf presence (treatment phase) and absence (pre- and post-phases) in home ranges and pastures, respectively, in southwest Alberta Canada. (0.16 MB PDF) [file pone.0011954.s001.pdf]

Table S1. Equations and illustrations of the habitat and movement metrics calculated from elk and cattle GPS-telemetry data during periods of wolf presence (treatment phase) and absence (pre- and post-phases) in home ranges and pastures, respectively, in southwest Alberta Canada.

Metric	***Equation***	***Illustration***
Speed (meters/hour)	**Eq. 1** $MR_i = \frac{d_i}{T_i}$ *, where i is the phase,* d_i *is the path length traveled during phase i, and* T_i *is the duration of the phase i.*	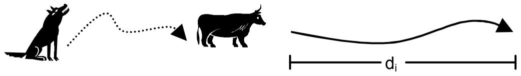
Sinuosity	**Eq. 2** $SN_i = \frac{d_{net_i}}{d_i}$ *, where* d_{net_i} *is the net displacement during phase i.*	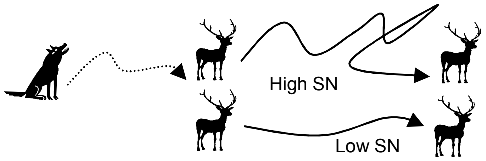
Average distance to Neighbours (meters)<br>*Cattle only*	**Eq. 3** $DN_i = \sum_{r_i=1}^{R_i} \frac{\left(\frac{\sum_{c=1}^{n_{ir}} \sqrt{(x_r - x_{cr})^2 + (y_r - y_{cr})^2}}{n_{ir} - 1} \right)}{R_i}$ *, where x and y are the UTM coordinates of animal locations, r is an animal location, R is the total number of animal locations (r) during phase i, c is the location of a neighbouring animal in the same pasture,* n_f *is the number of cattle in the same pasture,*	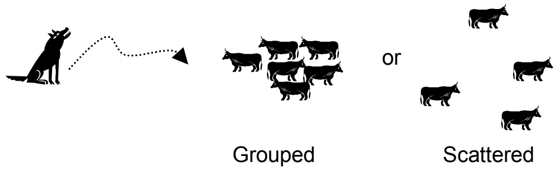
Percentage of Head Down<br>*Cattle only*	**Eq. 4** $HD_i =$ *percentage of the time spent Head Down during phase i,*	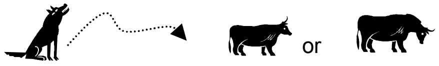

Terrain Ruggedness Index	**Eq. 5** $TRI_i = \frac{\sum_{r=1}^{R_i}\sqrt{\sum_{x'=x-1}^{x+1}\sum_{y'=y-1}^{y+1}(z_{x'y'} - z_{xy})^2}}{R_i}$ *, where* z_{xy} *is the elevation associated with location r,* $z_{x'y'}$ *is the elevation of the eight adjacent pixels in the DEM, and R is the total number of animal locations (r).*	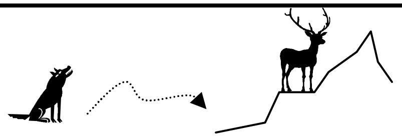
Slope (percent)	**Eq. 6** $SL_i =$ *average of the slope value for each animal location obtained during phase i.*	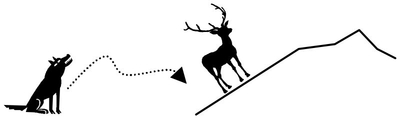
Distance to Forest Cover (meters)	**Eq. 7** $FC_i =$ *average distance between each animal location and the nearest patch of forest cover during a phase i.*	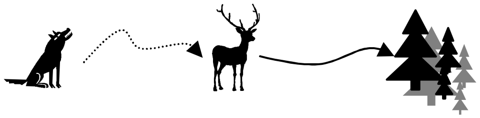
